# Claims-based Frailty indices may function as long-term risk estimates for elderly patients after hospitalization

**DOI:** 10.1016/j.lanepe.2021.100211

**Published:** 2021-09-06

**Authors:** Meral Kayikcioglu

**Affiliations:** Ege University School of Medicine, Department of Cardiology, Izmir, Turkey

The assessment of frailty has become an area of interest for researchers and clinicians in the recent years. Briefly, frailty is an age-dependent clinical syndrome characterized by increased vulnerability to stressors due to reduced functional reserve [1,2]. Frailty predicts the risk of hospitalization and mortality and might be used to estimate the utilization of healthcare by the aging population [1,2]. However, frailty assessment is underused due to the complex and time-consuming nature of the assessment tools. Moreover, there is no consensus regarding how frailty should be evaluated as the available measures exhibit marked inconsistency [2,3].

In their recent work in The Lancet Regional Health – Europe, Kundi and colleagues [4] provide insight to the use of administrative claims-based frailty indices for three acute conditions including acute myocardial infarction (AMI), heart failure (HF), and pneumonia. They have tested the performance of two established indices, Hospital Frailty Risk Score (HFRS) and the Johns Hopkins Claims-Based Frailty Index (JHCBFI) against endpoints of all-cause long-term (3-year) mortality, 1-year readmission, and 1-year mortality [5,6]. This nationwide analysis of 200,948 patients aged ≥ 65 years showed that readmission and mortality gradually increase with the frailty scores in patients presenting with AMI, HF, or pneumonia. The two indices varied in terms of discriminatory performance; JHCBFI showed a better discrimination for predicting mortality, while HFRS had a better discrimination for readmissions. Of note, both indices are entirely based on International Statistical Classification of Diseases (ICD) Codes that provide easier, low-cost, systematic evaluation of frailty. HFRS uses 109 different ICD-10-codes and is validated in elderly aged ≥ 75 years who had an acute hospital admission [5]. In the present work, Kundi et al extend the age range of HFRS to ≥ 65 years. Meanwhile, JHCBFI covers 21 criteria identifiable from ICD-9-codes validated for those aged ≥65 years [6]. Kundi and colleagues have appropriately transformed the ICD-9-codes to ICD-10 for the analysis of JHCBFI.

The retrospective nature of the study, the lack of information about the causes of death and disease severity, and lack of analysis of outcomes other than mortality and re-admission are the major limitations as acknowledged by the authors. Likewise, frailty indices based entirely on ICD-codes might be prone to measurement errors due to regional variations in coding of diagnoses. In fact, accuracy of the diagnosis codes is a matter of concern for all entirely claims-based studies. However, authors have obtained all ICD-codes assigned to patients within the 2-year period prior to index hospitalization from the ‘e-Pulse’ to minimize the missing ICD-codes. Of note, ‘e-Pulse’ is a personal health recording system maintained by the Turkish Ministry of Health integrating all information systems of all health institutions covering lab results, medical images, prescription, and medication details [7,8]. Therefore, ‘e-pulse’ contains clinical data besides administrative claims and integrated with the national death reporting system enabling the capture of accurate outcomes without missing data. Thus, these results may not be generalized to other countries. Indeed, country-specific frailty outcomes should be interpreted in the context of the characteristics and dynamics of elderly population studied. Though the elderly (≥65 years) constitutes 9% of Turkey's population, half of the health expenditures are attributed to this age group [9]. Turkey, like many countries, is facing the challenges of a growing elderly population with a prominent increase in the proportion of frail elderly [9]. However, traditional lifestyle and social structure of Turkey significantly decrease the social isolation of the elderly which is an advantage against frailty. Fundamental health reform was introduced in 2003 under the ‘Turkish Health Transformation Program’. The program aims to improve health by ensuring wider accessibility of healthcare to all citizens through a universal governmental insurance system [8]. A Multisectoral Action-Plan launched against non-communicable diseases in the last decade, has also improved healthcare for the elderly by integrating elderly health services into the family medicine system associated with home healthcare services [8,9]. Very recently, frailty index assessment has been integrated to ‘e-pulse’ enabling the risk evaluation of those aged ≥ 65 years both in primary and secondary care settings. And accurate claims-based frailty assessment at national level might further improve elderly health and help in developing more rational health policy, by facilitating the serial evaluation of hospital and health care services’ performance in terms of value-based care and payment [2].

Another limitation of the current study is that the follow-up period covers both a pre-pandemic and pandemic period, however further sensitivity analysis showed robust results also when excluding the pandemic data [4]. The fundamental impact of the pandemic and COVID-19 on frailty and outcomes of the elderly might compromise the validity of findings. Unfortunately, all outcome studies that were underway prior to pandemic potentially face this limitation. Thus, certain guidelines like the recent CONSERVE statement for studies requiring modification due to pandemic should be urgently implicated [10]. Furthermore, some frailty indices may be superior in predicting outcomes regarding COVID-19 pandemic which awaits further research.

In conclusion administrative claims constitute an alternative source of data by which frailty might be more easily assessed. Kundi and colleagues provide evidence for the use of entirely ICD-codes derived frailty indices for long-term risk prediction in elderly hospitalized for AMI, HF, or pneumonia at national-level which also might enable the evaluation of hospital and/or healthcare performance. However, the accuracy and reliability of the claims-based frailty indices depend on the health information technology infrastructure and completeness of documentation by healthcare providers [2].

## Contributors

MK as sole author contributed to all aspects of this manuscript

## Declaration of interests

Meral Kayikcioglu has received honoraria (for lectures and consultancy) from Abbott, Actelion, AstraZeneca, Abdi Ibrahim, Aegerion, Bayer Schering, Menarini, Sanofi Genzyme and Pfizer, and research funding from Aegerion, Amyrt Pharma, Amgen, Pfizer, and Sanofi, and has participated in clinical trials with Amgen, Bayer Schering, Sanofi, and Medpace for the last 3 years.
